# Distribution and conservation of species is misestimated if biotic interactions are ignored: the case of the orchid *Laelia speciosa*

**DOI:** 10.1038/s41598-020-63638-9

**Published:** 2020-06-12

**Authors:** Mayra Flores-Tolentino, Raúl García-Valdés, Cuauhtémoc Saénz-Romero, Irene Ávila-Díaz, Horacio Paz, Leonel Lopez-Toledo

**Affiliations:** 1 Instituto de Investigaciones sobre los Recursos Naturales, Universidad Michoacana de San Nicolás de Hidalgo, Av. San Juanito Itzícuaro s/n, Col. Nueva Esperanza, Morelia, Michoacán, CP 58330 Mexico; 2 CREAF, Universitat Autónoma de Barcelona, E08193 Bellaterra (Cerdanyola del Vallés), Catalonia, Spain; 3 Facultad de Biología, Universidad Michoacana de San Nicolás de Hidalgo, 48020 Morelia, Michoacán, Mexico; 4 Instituto de Investigaciones en Ecosistemas y Sustentabilidad, Universidad Nacional Autónoma de México Unidad Morelia, Antigua Carretera a Pátzcuaro, 8701 58190 Morelia, Michoacán, Mexico; 5 Universitat Autónoma de Barcelona E08193 Bellaterra (Cerdanyola del Vallés), Catalonia, Spain

**Keywords:** Ecological modelling, Ecological networks, Conservation biology

## Abstract

The geographic distribution of species depends on their relationships with climate and on the biotic interactions of the species. Ecological Niche Models (ENMs) mainly consider climatic variables only and may tend to overestimate these distributions, especially for species strongly restricted by biotic interactions. We identified the preference of *Laelia speciosa* for different host tree species and include this information in an ENM. The effect of habitat loss and climate change on the distribution of these species was also estimated. Although *L. speciosa* was recorded as epiphyte at six tree species, 96% of the individuals were registered at one single species (*Quercus deserticola*), which indicated a strong biotic interaction. We included the distribution of this host tree as a biotic variable in the ENM of *L. speciosa*. The contemporary distribution of *L. speciosa* is 52,892 km^2^, which represent 4% of Mexican territory and only 0.6% of the distribution falls within protected areas. Habitat loss rate for *L. speciosa* during the study period was 0.6% per year. Projections for 2050 and 2070 under optimistic and pessimistic climate change scenarios indicated a severe reduction in its distribution. Climaticaly suitable areas will also shift upwards (200–400 m higher). When estimating the distribution of a species, including its interactions can improve the performance of the ENMs, allowing for more  accurate estimates of the actual distribution of the species, which in turn allows for better conservation strategies.

## Introduction

Ecological niche models (ENMs) are useful tools for predicting the potential spatial distribution of species. Such models have been applied in multiple studies to assess contemporary and future species habitat availability, as well as the impact of environmental change and anthropogenic factors, such as fires and land use change, on the suitable distribution of species. Thus, ENMs can be used to identify priority areas for conservation^1–4^. These models generally use occurrence data and environmental variables to estimate the range of suitable environmental conditions for each individual species^5^. This method usually considers only environmental factors influencing the species distribution, while ignoring others, such as the interaction with other species^6^. In general, the distribution of a species is the consequence of several factors, and sometimes also of the interaction between them^7^. The three most important drivers of species distributions are: 1) the abiotic environment, 2) the biotic environment and 3) the accessible space. The combination of these drivers allows the estimation of the distribution of a species^5^. The abiotic environment and biotic interactions limit the species ability to persist in an area. For example, the climatic and edaphic conditions impose physiological limits^7^, while the biotic interactions affect the species distribution through mechanisms such as predation, competition, parasitism, mutualism or commensalism. For some species these biotic interactions play a very important role at local extents, but in general these relationships at larger geographic scales have been dismissed. Thus, most of the ENM studies assumed that biotic interactions are unimportant in the determination of the species geographic ranges^8–10^. However, several studies have demonstrated that the inclusion of biotic interactions in ENMs improves the performance of these models, both at local and regional scales^1,9,11–13^.

Epiphyte plants are a group that exemplifies the importance of biotic interactions. Such species establish and complete their entire life cycle on the bark of trees, and therefore the distribution of their host tree species strongly influence the distribution of these plants^8,14^. However, despite the essential role that biotic interactions play in determining the distribution of some species, very little is known about the functioning of these ecological relationships^5,8,15^. Moreover, the importance of the interactions that occur among species at the macro-scale is still subject of debate^8,16^.

It is  expected that climate change will modify the range of an important number of species^17–20^, which may modify the composition of local communities, and may create transient communities that could be dominated by generalists species^21–23^. To understand the possible effects of climate change on species distributions and ecosystem relationships, and to promote biodiversity conservation, it is essential to consider all the important drivers of the distribution of species when projecting future changes^24^. For species that are highly dependent on biotic interactions, it is necessary to understand the effect of these interactions on their distributions^25,26^. For example, currently interacting species may no longer occupy the same areas in the future as a result of climate change, because they could migrate at either different speeds or directions^26–28^. Some biotic interactions, such as predation, competition and mutualism, are especially important for the maintenance of biodiversity^29,30^, and they can be decisive in determining the response of interacting species to climate change^11^. Biotic interactions are key for evolutionary and ecological processes and mediate the responses of some species to climate^21^. Similarly, climate can affect the direction, frequency and intensity of the interactions. Thus, understanding the complexity of the relationship between climate and biotic interactions is essential in order to predict the future habitat distribution of some species, especially those strongly dependent on these interactions.

The establishment and abundance of epiphytic plants is largely determined by the microclimatic conditions provided by their host tree, like a protecting shadow and support well above the ground^31,32^. This host-guest relationship therefore largely determines the distribution of these epiphyte species^32^. Future changes in the distribution of host species due to climate change may thus act to reduce the distribution of epiphytes^11,33^. Moreover, these species live in tropical forests, a habitat under particular threat due to changes in both climate and land use^34^. Finally, these species are very sensitive to variations in temperature and precipitation, since they obtain the nutrients and water they require directly from the surrounding air^35,36^. For this reason, epiphytes are considered good bioindicators^35–37^.

Orchidaceae is one of the most diverse families of all vascular plants in the world. With over 1300 species, many of them endemic, Mexico is not an exception for its diversity and endemism^38^. Orchids are widely distributed in all major Mexican ecosystems, mostly on humid biomes, and exhibit a great diversity of life forms, including epiphytes, terrestrial and lithophytic species. They have also developed amazing biotic interactions, such as very specific mycorrhiza and pollination. Flowers of orchids are one of the most fascinating and beautiful in the whole plant kingdom, which make them very attractive for collectors. In Mexico, many species are extensively collected for religious and cultural celebrations (e.g. Day of the Dead), and they represent an economically valuable non-timber forest product, which contributes to the livelihood and welfare for low-income people living in or near to forests. Due to a still important land use change (from forest to agricultural or grazing) and illegal harvesting, orchids in Mexico are vulnerable to extinction and many species are considered endangered^38–40^. One of these endemic species is *Laelia speciosa*, which is an epiphytic orchid that is particularly appreciated for its ornamental and cultural value in Mexico. In Michoacán state (central West México), it is estimated that about 2,500 reproductive individuals are collected annually^39^. This has led the species to be classified as under high conservation risk^38,41,42^. In the present study, we used this threatened orchid to study the importance of considering host tree distribution for the accurate assessment of the distribution and conservation of the species. Thus, we addressed the following questions: i) how important is to consider the distribution of host species for modeling the distribution of *L. speciosa*? and ii) what are the consequences of using biotic variables for modeling the influence of climate change on the distribution of *L. speciosa*? Finally, we discussed the protection status of *L. speciosa* and contemporary and future factors that endanger its conservation.

## Methods

### Study species

**Figure 1:**
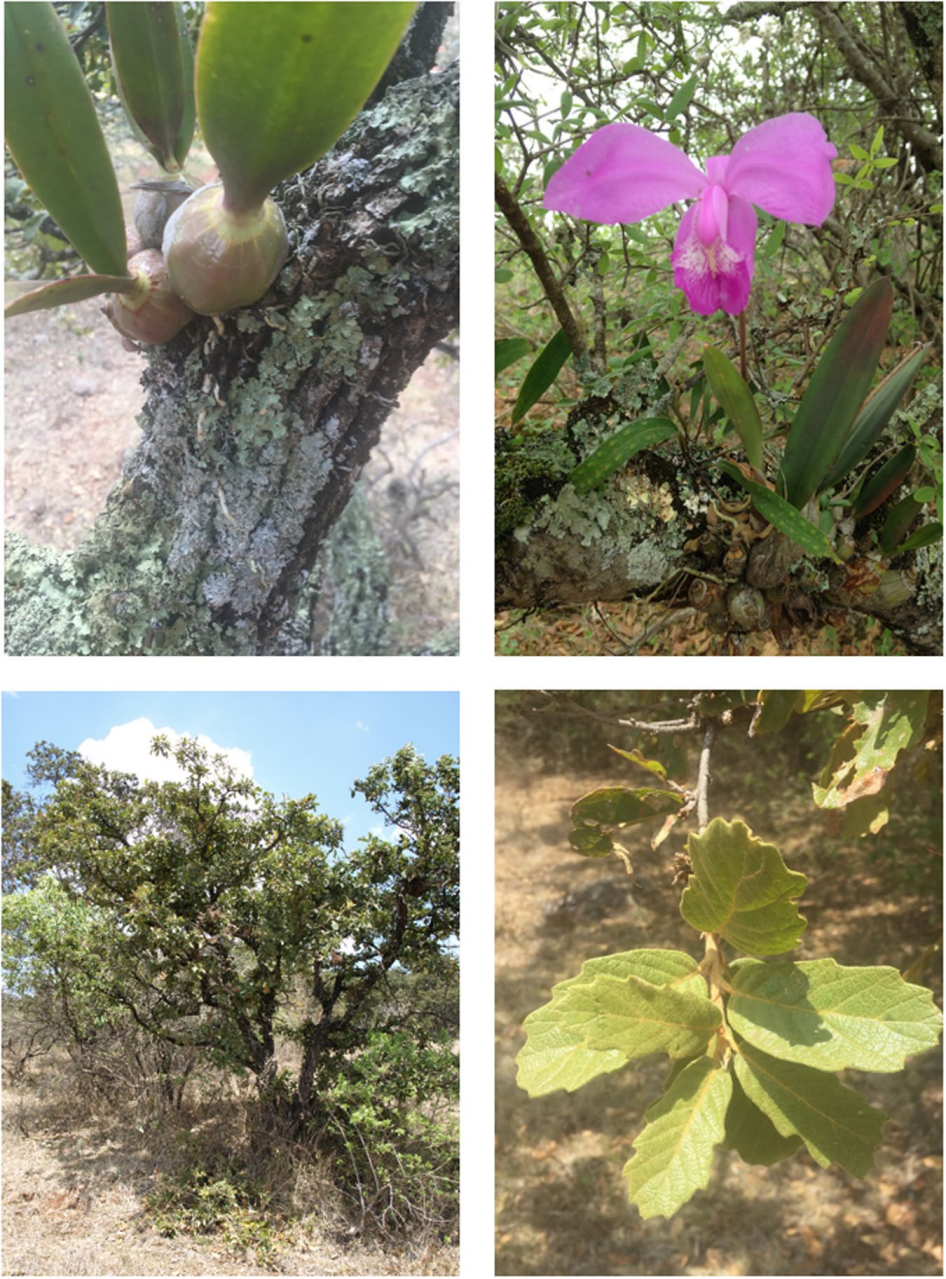
*Laelia speciosa* pseudobulbs and flower (top) on the bark of *Quercus deserticola* (bottom), one of the hosts.

*Laelia speciosa* is an epiphytic orchid, which grows simple or forming compact clumps of 12–40 cm high (Fig. 1). Pseudobulbs are subglobose to ovoid, and slightly compressed (3–6 cm high and 15–40 wide). It presents one-two lanceolate leaves 8–16 cm long. Flowers are large and showy (10–16 cm diameter) with pink to lilac-purplish color. It is endemic to Mexico, and epiphytic in *Quercus* species, mainly in temperate forests (Fig. 1) at altitudes ranging from 1900 to 2500 m, with a mean rainfall and temperatures of 850 mm and 17.5 °C, respectively^42^. *L. speciosa* is widely collected for its cultural and ornamental value, mainly associated with its beautiful flowers. This has led the species to face serious conservation problems^38,41,42^. Although, the species is not included in the IUCN red list^43^, the Mexican conservation list considers it as a species under Special Protection^40^.

### Identification and preference of hosts

To investigate the importance of species interactions in the distribution of *L. speciosa*, we considered the host tree species as a proxy of a highly important biotic interaction (probably commensalism or facilitation) that could determine the distribution of the species. To identify the preference of *L. speciosa* for a particular host tree, we sampled twelve localities covering the entire range of distribution for the species. We selected landscapes dominated by forest cover: five localities in Michoacán, two in Jalisco and in Hidalgo, and one each in Guanajuato, Aguascalientes and Durango. At each locality where the presence of *L. speciosa* was recorded, a random 20 × 20 m quadrat was established. Within each quadrat, we quantified all trees and shrubs >1 cm diameter at breast height and recorded their taxonomic identity and the presence/absence of *L. speciosa*, and registered the number of individuals. All tree species were taxonomically identified and vouchers were deposited at National Herbarium of Mexico (MEXU). The number of *L. speciosa* individuals on each tree species recorded and the percentage of host trees with *L. speciosa* presence was calculated. This information was used to weight the most important host tree species model, which was then included in the modeling of *L. speciosa*.

### Presence information

The presence information of *L. speciosa* and the host tree species used for modeling were obtained through a meticulous review of seven Mexican Herbaria: Asociación Mexicana de Orquideología (AMO), Herbario Nacional de México (MEXU), Instituto Politécnico Nacional (ENCB), Instituto de Ecología-Pátzcuaro (IEB-Pátzcuaro), Universidad de Guadalajara (IBUG), Centro Interdisciplinario de Investigación para el Desarrollo Integral Regional (CIIDIR-Durango) and Universidad Michoacana de San Nicolás de Hidalgo (EBUM). We considered only those records with complete information regarding locality and obtained 96 unique presence records for *L. speciosa*. To avoid spatial autocorrelation among presence points, we conducted a pattern analysis following Hengl^44^ and finally yield 66 unique presence points (see details in SI1).

### Ecological Niche Modeling

The climate information used in the modeling was obtained from the WorldClim platform (http://www.worldclim.org/), and included 19 independent variables derived from temperature and precipitation that have been widely used in modeling studies^45,46^. From these variables, a group was selected considering three criteria: i) expert knowledge, ii) importance of the variables for the distribution of *L. speciosa* and its hosts and iii) non-correlated variables. For the second criterion, we used the percentage contribution obtained from a previous exploratory model in Maxent ver. 3.4.1^47^ and included all of the variables that contribute at least 1%, and together contribute 95%, to explanation of the variance. For the third criterion, we used a matrix correlation in ENMTools and considered all variables with a correlation coefficient of <0.8^48^. For the whole list of variables used in the modeling of *L. speciosa* and the host tree species, see supplementary information. The limit of the biogeographical regions with the presence of *L. speciosa* and *Quercus deserticola* was determined in order to more accurately represent the accessible area (“M” in the BAM diagram *sensu* Soberón & Peterson^7^) that can be occupied by a given species^7,49^.

To model habitat suitability of *L. speciosa*, we used the program Maxent version 3.4.1.^47^ which has demonstrated good performance in the projections of current and future climate habitat distributions^50,51^. This program only requires presence data^3,52–55^. We run the models using the 75% of the presence data and 25% for validation with the following parameters: maximum iterations (500), a convergence threshold (0.00001) and the maximum number of background points was 10,000. In order to avoid overfitting of the test data, we set the regularization multiplier value as 1. The predictive accuracy of the models was evaluated by a 10-fold cross-validation.

To evaluate the importance of host tree species to the distribution of *L. speciosa*, we used four different methods: i) an abiotic model (ACLIM), which included only the selected climatic variables. All of the other models included the same climatic variables, plus a layer of a presence model of the host trees species. This layer of the weighted presence model was included to produce the other  three models: ii) a biotic continuous model (BCONM), including the continuous presence of the host tree species model (previously obtained using only climatic variables), iii) a biotic categorical model (BCATM), which included the host tree species information, but with binary (presence/absence) categorization, and iv) a biotic strict model (BSTRM), which only included the localities of presence of the host tree species.

For both *L. speciosa* and the hosts, the output format of the model was logistic and then transformed into binary predictions, considering the maximum test sensitivity plus specificity threshold, which has been used effectively in previous studies to produce precise predictions^56,57^.

## Analysis

To validate the different models and select the optimum, we used four different validation methods: i) AUC (area under the ROC curve)^58^, ii) a binomial test to evaluate if the model is better than one produced at random^59^, iii) partial ROC (receiver operating characteristic), which provides a robust prediction^60–62^ and iv) the True Skill Statistic (TSS)^63^. The model with the best performance was used to obtain the historic and then the current distribution model of *L. speciosa*.

### Climate change scenarios

The climate change projections were made using two models of intensity of increase in carbon emissions: RCP2.6 (a very optimistic model) and RCP8.5 (pessimistic model, although perhaps realistic, given the contemporary trend of greenhouse-effect gas emissions), by the years 2050 and 2070, of the Coupled Model Intercomparison Project - Phase 5 (CMIP5). RCP2.6 considers low levels of CO_2_ emissions and assumes that annual emissions of greenhouse gases will reach up to 3.0 Wm^−2^ of radiative forcing and then decrease to 2.6 Wm^−2^ by 2100, while RCP8.5 assumes that emissions will continue to increase throughout the 21st century, reaching 8.5 Wm^−2^ by 2100^64^. These projections were also made in the MaxEnt 3.4.1. For each of the four climate change scenarios (RCP2.6–2050, RCP8.5 2050, RCP2.6–2070 and RCP8.5–2070), two types of models were produced: i) an abiotic model, which only considers climatic variables and ii) a biotic model that, in addition to the climatic variables, also considers the future projection of host species as an additional layer. This layer is the probabilistic model of the most preferred host tree species, which was weighted based on *L. speciosa* preference. Multivariate environmental similarity surface (MESS) analyses were conducted to examine where analog (similar) versus non-analog (novel) climate exists in geographic space of the projected models^65^.

For the biotic models, we assumed that the current *L. speciosa* preference for hosts will be maintained in the future. The logistic outputs of *L. speciosa* were processed in ArcGis 10.2 and were categorized as binary (0 or 1) using also the maximum training sensitivity plus specificity threshold^56^, which was used for the contemporary models. This threshold maximizes the sum of sensitivity and specificity compatible with the three solid principles for threshold selection (objectivity, equality and discriminability criteria)^57^.

The current and future models were compared using the minus tool of *Algebra of maps* in the program ArcGis 10.2^66^. The resulting layers were analyzed in order to obtain the net and specific changes. The net changes were quantified in the loss or gain of total area of distribution, while the specific changes were those relative changes in the comparison of pixels of presence and absence between current and future models.

## Results

*Laelia speciosa* showed a marked preference for *Quercus deserticola*, as 96% of the recorded individuals were hosted by this species. Ninety percent of the *Q. deserticola* trees had at least one individual of *L. speciosa*. However, the species was also present on another eight tree species (*Q. praeco*, *Q. laeta*, *Q. glaucoides*, *Q. castaneae, Q. obtusata. Q. grisea, Opuntia sp*. and *Ipomoea murocoides*).

### Contemporary ecological niche modeling

**Figure 2:**
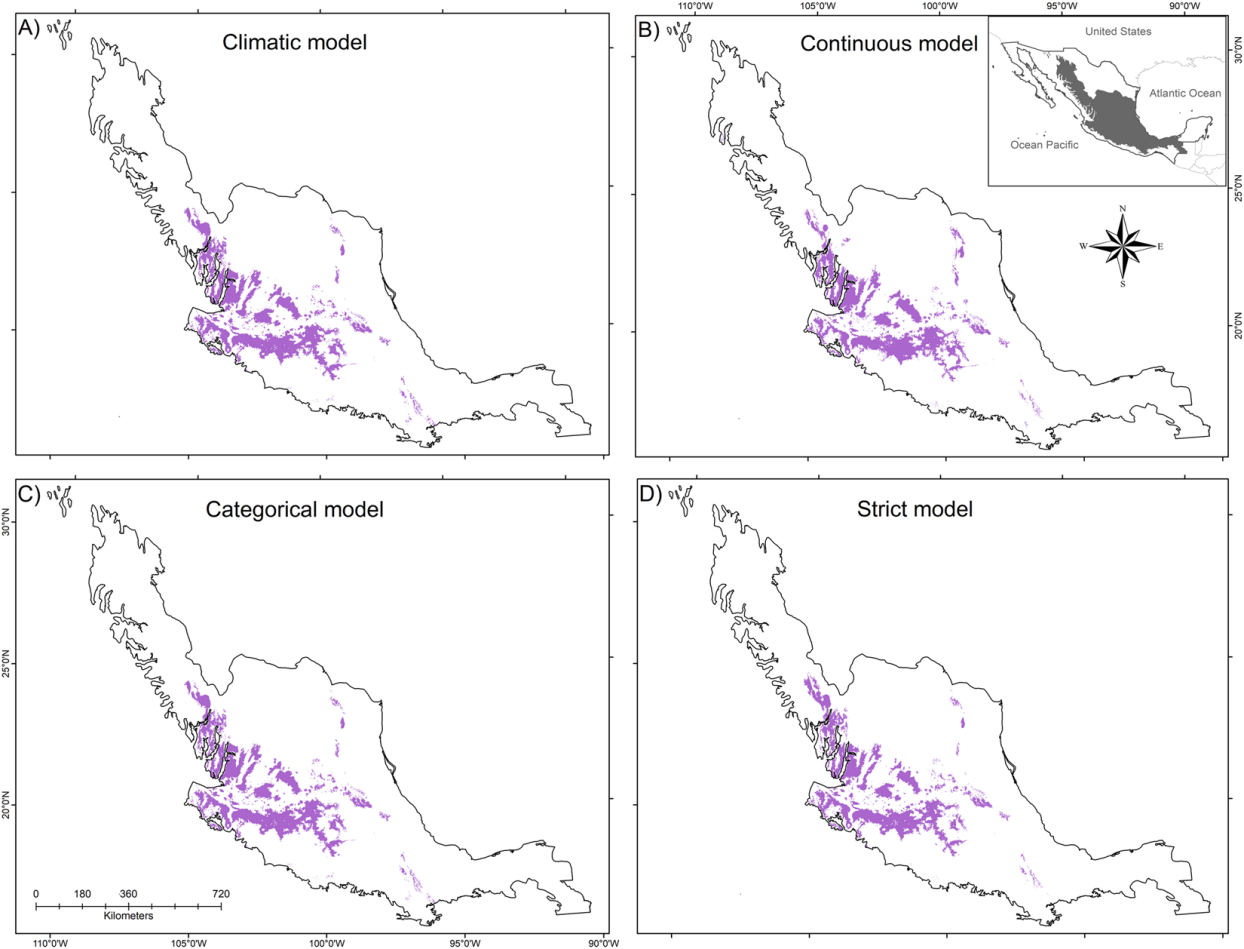
Distribution models for *Laelia speciosa* considering climatic variables and presence of the most important host species (*Quercus deserticola*). (**A**) Climatic model (ACLIM), (**B**) Continuous model (BCONM), (**C**) Categorical model (BCATM) and (**D**) Strict model (BSTRM). See Table 1 for further details.

We found that the inclusion of the main host tree species *Quercus deserticola* in the modeling process improved significatively the performance of the models. Specifically, the model BCONM that included the continuous probability of presence of *Q. deserticola* presented the highest performance measures and the climatic model (ACLIM) generally resulted in lower performance (Table 1). The strict model (BSTRM) produced the lowest historic distribution area of presence in Mexico (3.9%), with 76,299 km^2^, which represents 8,000–3,000 km^2^ less than the other models (Fig. 2; Table 1). This area is found in 24 states, with 76% found in five states (Jalisco, Michoacán, Guanajuato, Zacatecas and Durango). Since the continuous model presented the optimum performance, we used it for the rest of the analysis.

**Table 1:** Performance measures of the model applied to climate models and considering the interaction with the host (*Quercus deserticola*).

Model	AUC	Binomial test	Partial ROC	TSS	Occupied area (km^2^)	Occurence (%)
ACLIM	0.965	0.75 < 0.03	1.73	0.920	84,302.2	4.3
**BCONM**	**0.971**	**0.81** < **0.01**	**1.74**	**0.931**	**79,903.8**	**4.1**
BCATM	0.966	0.75 < 0.03	1.73	0.924	82,476.0	4.2
BSTRM	0.968	0.75 < 0.03	1.73	0.921	76,298.6	3.9

Evaluation methods: Area Under the Curve (AUC), Binomial Test, Partial ROC (receiver operating characteristic) and the True Skill Statistic (TSS). Abbreviations for models: BCONM = biotic continuous model, BCATM = biotic categorical model, BSTRM = biotic strict model and ACLIM = abiotic/climatic model. Model in bold was the one with best performance in all tests.

**Figure 3:**
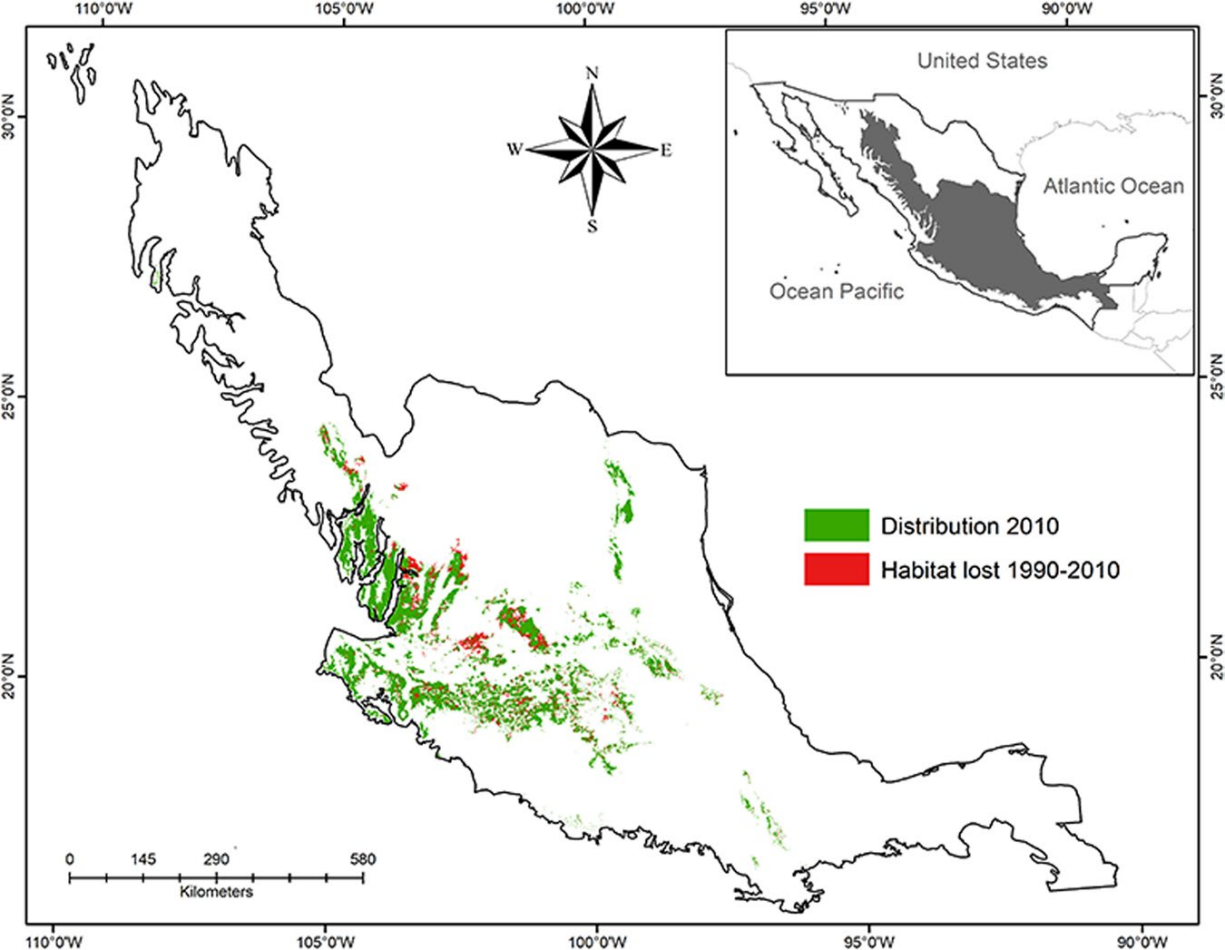
Distribution of *Laelia speciosa* and habitat lost between 1990–2010.

**Figure 4:**
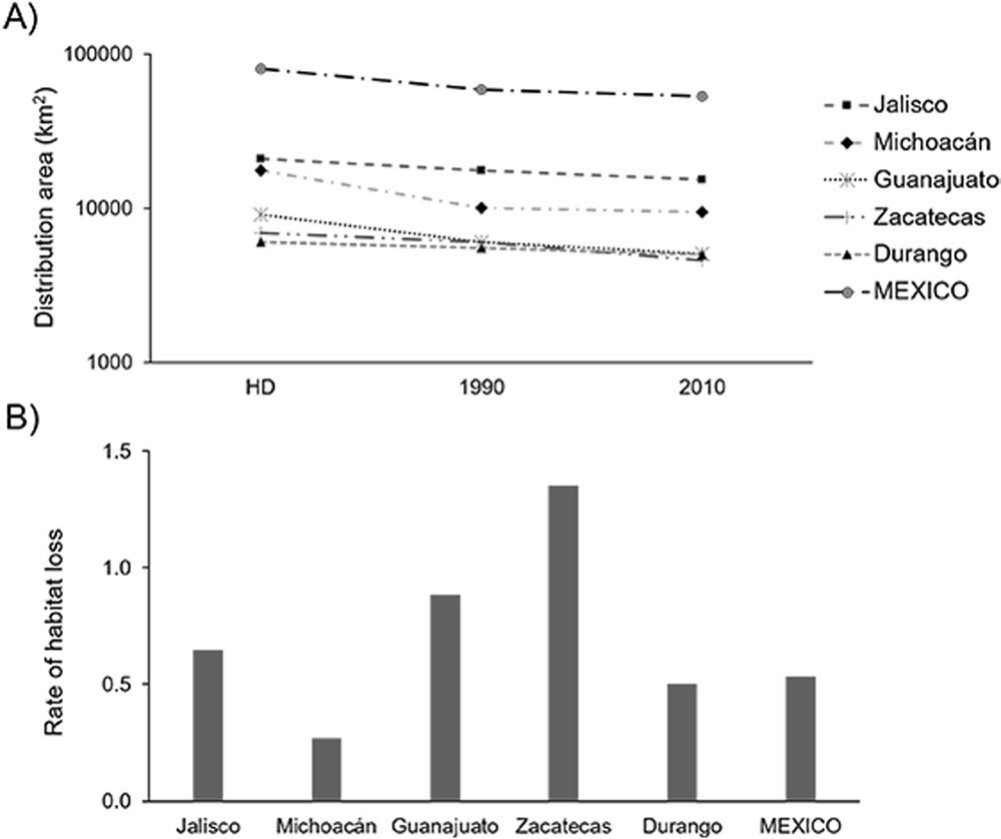
Distribution of *Laelia speciosa* for the five most important states and for whole country (Mexico). (**A**) reduction in distribution and (**B**) rate of habitat lost.

The continuous model (BCONM) indicated that the 1990 habitat distribution of *L. speciosa* in Mexico was 58,861 km^2^, which represents 73.7% of the historic suitability. In the 1990–2010 period, this area decreased by a further 10%, reaching an area of 52,892 km^2^, which represents 2.7% of the land surface area of Mexico (Fig. 3). The distribution area also changed among states during these periods: for the period 1990–2010, the state that lost the largest area was Zacatecas, decreasing from 6,045 to 5,059 km^2^ and representing the highest loss of distribution area (23.8%), while Michoacán had the lowest reduction, with 5.2% in the same period (Table 2). As a consequence, the rate of habitat loss of *L. speciosa* for Mexico was 0.53% (1990–2010) and important differences were presented among states, with Zacatecas and Michoacán presenting the highest and lowest rates, respectively (Table 2; Fig. 4).

**Table 2:** Distribution of historical, contemporary and future *Laelia speciosa* that it occupies in different states and throughout Mexico (total).

State	Distribution historical (km^2^)	1990 (%)	2010 (%)	PHL 1990–2010 (%)
Jalisco	20,885	83.6	73.4	0.65
Michoacan	17,509	57.0	54.0	0.27
Guanajuato	9,077	66.6	55.7	0.88
Zacatecas	6,939	87.1	66.4	1.35
Durango	6,009	92.2	83.4	0.50
Other	19,485	70.8	68.9	0.14
Mexico	79,904	73.7	66.2	0.53

The historical distribution is shown in km^2^ and the distribution in the following years (1990, 2010) as a percentage. In the last columns, the annual average percentage of habitat loss (PHL) for the period 1990–2010 is shown. In the “Other” category, the area of 19 states in which the species is distributed but in a smaller proportion is concentrated.

### Climate change scenarios

MESS analysis indicated regions climatically similar to the native niche in both models (climatic and biotic), except for northeast areas, that present non-analogous conditions to those present in the native range (Fig. SI 1: A,C). The mean temperature of the wettest quarter was the variable which present the most dissimilar values related to those areas (Fig. SI 1: B,D).

**Figure 5:**
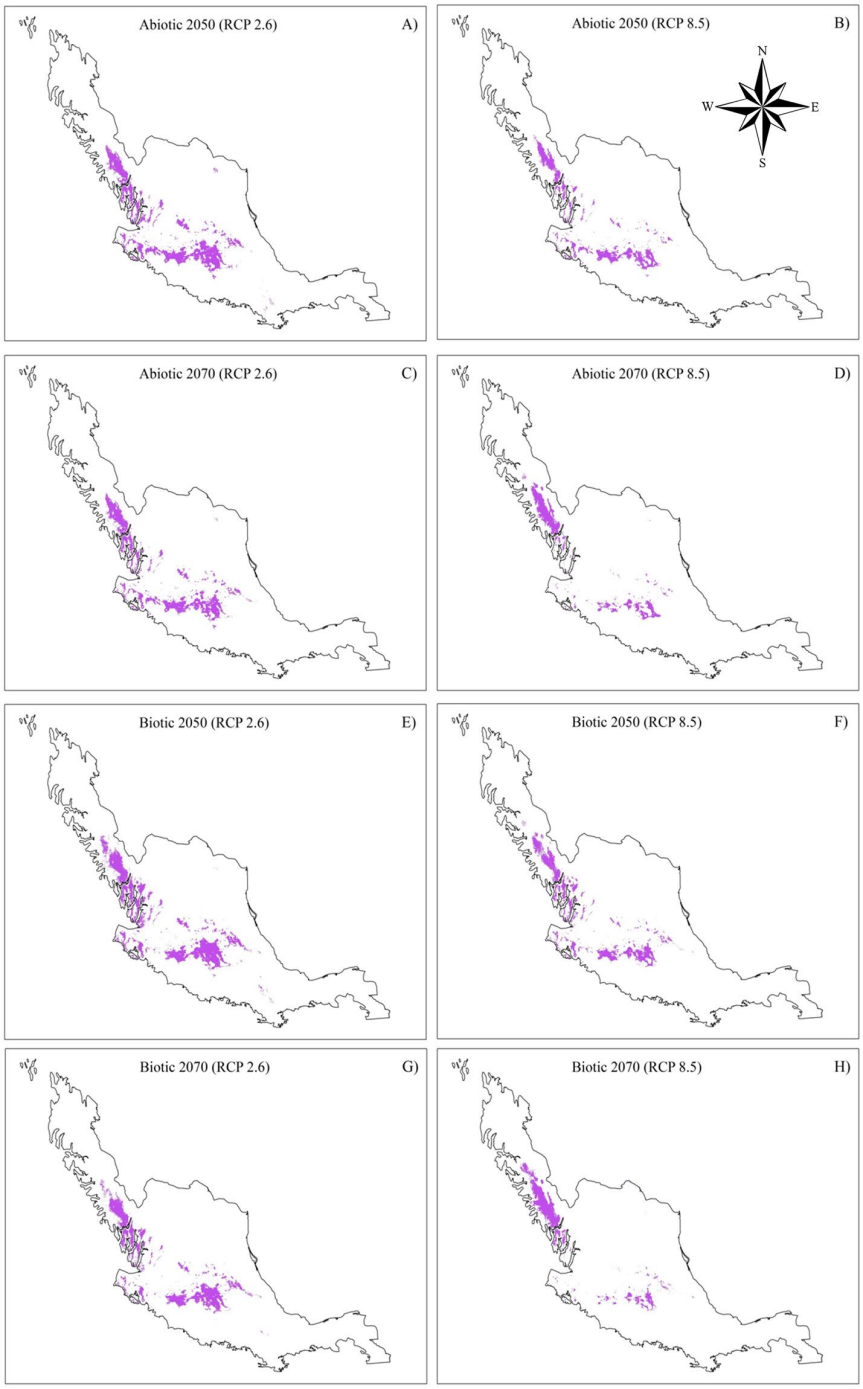
Distribution of *Laelia speciosa* under climate change scenarios for 2050 and 2070. (**A**–**D**) represent the abiotic scenarios and (**E**–**H**) are the biotic scenarios.

In the comparison of the current models (biotic and abiotic) with the optimistic models, the RCP2.6–2050 predicted a decrease between 22.6 and 31.7% in the distribution of *Laelia speciosa* (Fig. 5A,E). The scenarios changed when comparing the models with the RCP2.6–2070, in which the distribution area was further reduced, and the biotic model predicted a reduction of 38.3% while the abiotic model predicted a reduction of up to 43.2% (Fig. 5C,G).

The changes were even more pronounced when comparing the current distribution of *L. speciosa* with the pessimistic scenarios for both periods (2050 and 2070). The RCP8.5–2050 scenario predicted reductions of 48.8% (biotic) to 58.0% (abiotic), while the RCP8.5–2070 estimated area losses of 61.2 and 66.8%, respectively (Fig. 5D,H).

**Figure 6:**
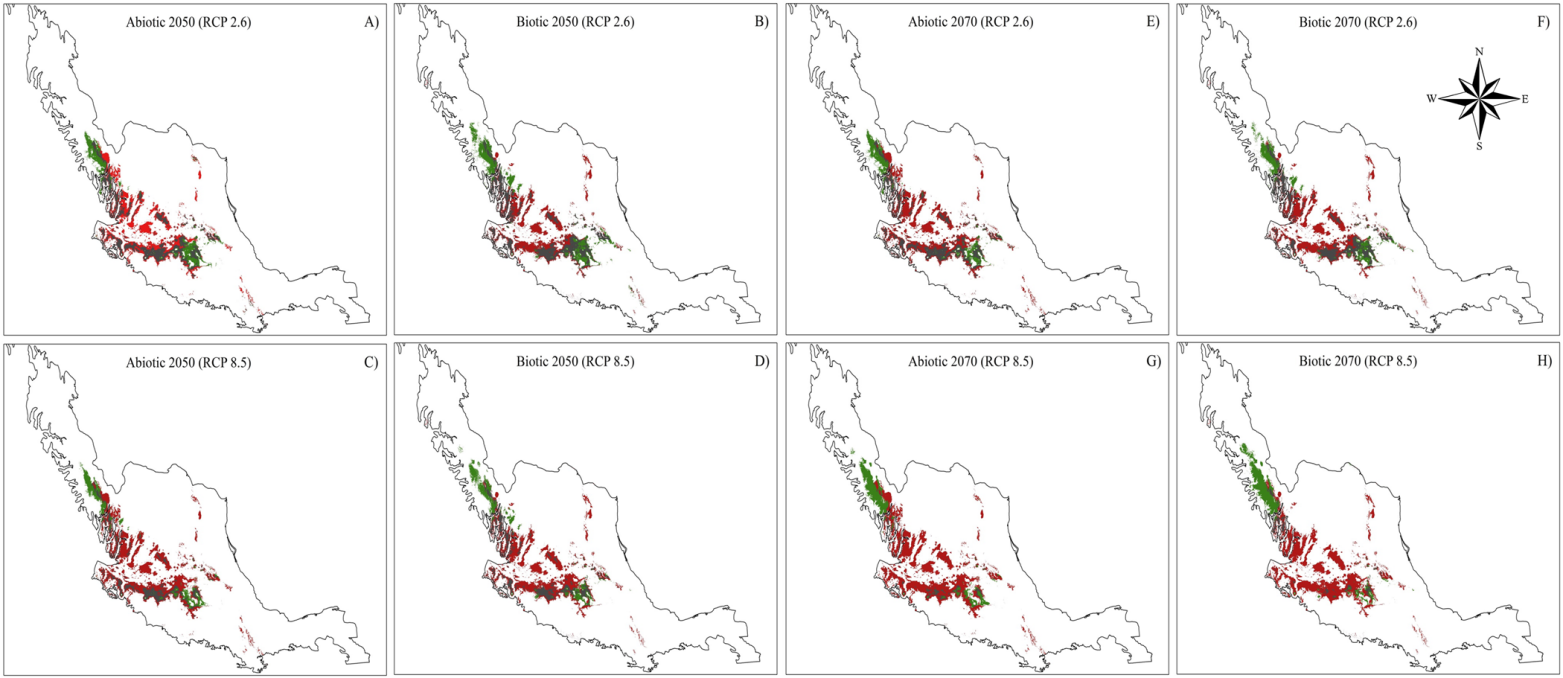
Comparisons between present and future models (2050 and 2070) of the distribution of *Laelia speciosa*. In green it shows the area that the species will potentially occupy (Relative gains + Permanence), in red the area that will be lost for (Risk of extinction) and in gray the area that will remain. (**A,B**) represent the optimistic scenarios, while (**C,D**) represent the pessimistic scenarios.

**Figure 7:**
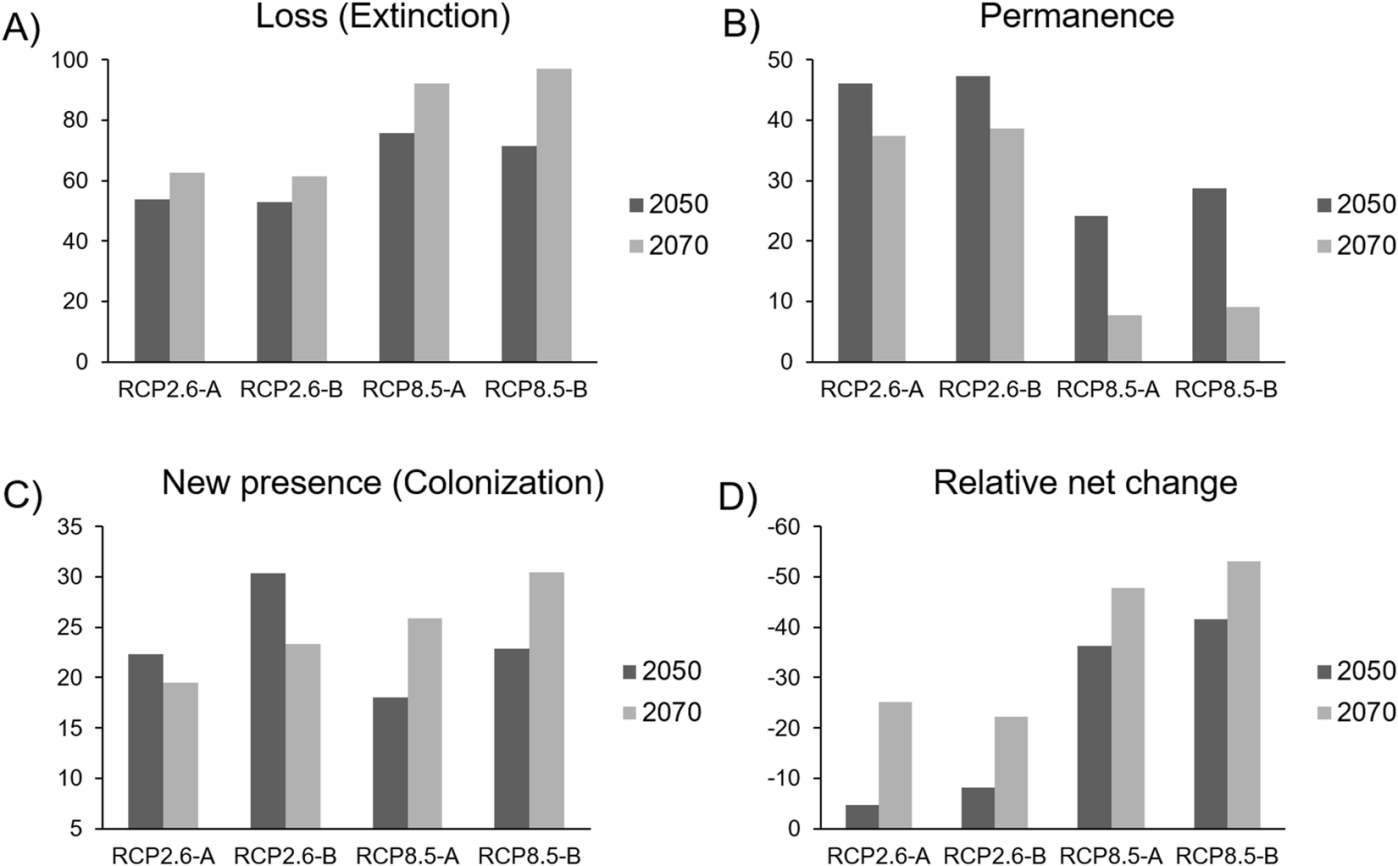
Comparisons of the abiotic models (only climatic variables) and biotic (climatic and host variables) of the current-future distribution of *Laelia speciosa*. Relative profits are new areas of distribution of *L. speciosa* in the future. Permanence: contemporary area that will remain in the future. Extinction risk: indicates those areas where the species is present and according to predictions will be lost in the future. Net relative change obtained by adding the relative gains plus permanence minus the contemporary distribution.

It is important to consider that these net changes differed when comparing the relative changes of permanence, gain (colonization) and loss (extinction) of area between current and future models. Based on the predictions of future models (Fig. 6), the original distribution areas will be considerably reduced. The projections of both the abiotic and biotic models, RCP2.6 (Fig. 6A,B,E,F) and RCP8.5 (Fig. 6C,D,G,H), foresaw reductions ranging from 52.7.6% by 2050 to 96.9% by 2070 (Fig. 7A). On the other hand, the models predicted areas of gain in the distribution that can be interpreted as colonization (Figs. 6 and 7C). All of the models predicted colonization ranging from 18.0% to 30.5%. Considering permanence, extinctions and colonization together, the relative net change was negative in all of the comparisons (Fig. 7D). In general, a reduction of 22.3–66.4% was obtained in all models relative to the current models.

### Conservation assessment

The suitability area of *L. speciosa* coincides with 37 protected natural areas (PNA) in Mexico, however its presence has been only validated at one Natural Area (Barranca de Metztitlán) in Hidalgo State. The distribution of the suitable area in that PNA covers 312 km^2^, which only represents 0.6% of the total distribution area in Mexico (Figure SI2).

According to our estimates of suitability area and population reduction, under the IUCN system and the Mexican red list^38^, *L. speciosa* can be classified as Vulnerable and Threatened, respectively, since the species has showed a loss in suitability area of 10% and it has been foreseen that its distribution will be affected to an even greater degree in the future, losing up to 70% of its current distribution in the next 100 years (Table 3).

**Table 3:** Conservation status of *Laelia speciosa* based on IUCN and the Mexican red list criteria.

Criteria	IUCN
A. Population reduction	10%
B. Geographic range	>20 000 km^2^
C. Adult individuals	>10 000
D. Restricted distribution	─
E. Quantitative analysis	≥20%
Category UICN 2014	─
Proposed category	*Vulnerable (VU)*
**Criteria**	**SEMARNAT (Mexico)**
A.- Amplitude of the distribution	3% (4)
B.- Habitat status	Intermediate 2)
C.- Vulnerability	Average (2)
D.- Anthropogenic impact	High (4)
Category SEMARNAT 2010	Special protection (Pr)
Proposed category	*Threatened* (A)

## Discussion

### Distribution under current scenarios

The ideal host trees for many species of orchids and bromeliads are those that present rough textured bark that can offer microenvironments for germination and establishment^67^. In the case of *L. speciosa*, we detected a higher preference for *Quercus deserticola*. This could happen due to the bark characteristics of this tree species (Fig. 1), such as pronounced roughness. However, in the same locations, we found other arboreal species with similar bark characteristics (*Q. praeco, Q. laeta* and *Q. glaucoides*) that had no presence of *L. speciosa*. This could be due to interactions with other intermediate species^38,68–70^. Epiphytic orchids have also a strong relationship with mycorrhizal fungi that interact with the host trees, and with other guest species through nutrient cycling^71,72^. Such relationships affect important processes in the orchid life cycle, such as germination and establishment. For example, Hernández-Apolinar^73^ found that 96% of *L. speciosa* individuals were associated with several lichen species of the genus *Parmelia*. It is therefore important to conduct further research on the role of lichens and fungi in determining the establishment of *L. speciosa* on host trees^8,9,11,74–76^.

Although many studies still ignore biotic interactions when modelling species distributions, we have showed here that including the distribution of a host species is of great relevance when modelling an epiphyt distribution. Our results indicate that the inclusion of information regarding the distribution of *Quercus deserticola* in ENMs was decisive for improving their performance. We believe that for very specialized guest species, it is essential to include host occurrence information when modeling their distribution, as environmental-only models may overestimate their distribution range^8,14,75,77^. This occurs because with environmental-only models the entire climatic range where the species occurs is considered to be suitable. In contrast, when host occurrence information is integrated into the model, the range of suitable conditions is adjusted to climatically suitable areas that also present hosts for the species^78^.

A few studies with ENMs have included biotic interactions in their modeling design. In general, these studies found that biotic relationships play a decisive role in predicting the range of distribution of the target species, and improved model performance. For example, Giannini *et al*.^75^, studied two groups of interactions (pollination and parasitism) in six bumblebee species (*Bombus*, Apidae) from the British Isles. They found that the inclusion of strong and specialized interactions, but not weak and generalist ones, is highly important for estimating the distribution of the studied species. Our findings support such claims for highly specialized epiphytes. We highlight not only the necessity of inclusion of biotic information, but also the relevance of the way in which it is incorporated, depending on the type of interaction considered^8,11,75,77,79^. In a commensalism interaction, as in the case of *L. speciosa*, including the probability of presence of the guest as a predictive variable improved the prediction of the model. This method allowed the model to integrate the climatic conditions offered by the host to the focal species.

Pearson and Dawson^80^ argue that biotic interactions only play an important role at fine resolution scales, but not at meso and macro scales, and conclude that climate alone is sufficient for predicting species distribution across large ranges. In contrast, Araújo and Rozenfeld^81^ show that interactions are also important at macroecological spatial scales, especially positive interactions such as mutualism and commensalism. For *L. speciosa*, we showed that inclusion of a strong biotic interaction improves model performance at large scales, and recommend the incorporation of host distribution information for epiphyte niche modeling.

### Distribution under climate change

The models under climate change scenarios predicted important reductions in the future habitat of *L. speciosa*, especially in projections for the year 2070. The scenario RCP8.5–2070 projects a 66.8% loss of the species distribution. For this period, increased values of CO_2_ (indicating a 8.5 watts per metre squared – W/m2 – forcing increase relative to pre-industrial conditions and temperature (2.0–3.5 °C)) are expected. If temperature increases as projected (3 to 6 °C by the end of the 21st century), the species will be severely affected. Climate change is likely to affect the metabolism, phenology and morphology of the species and will transpose their distribution ranges towards higher elevations^34,82–84^.

Our analysis also indicates that some suitable areas will be lost under these climate change scenarios, while others will be maintained or gained. The areas with the highest risk of extinction are those found at the center of the North-South current species distribution (Neovolcanic axis and southern Altiplano). The high risk of extinction in these areas may be related to the orography that characterizes these regions. In these areas, the mountains do not exceed 2600 m.a.s.l., which is the current upper altitudinal limit of *L. speciosa* distribution. Considering that the ideal conditions for *L. speciosa* in the future will be found at higher altitudes, the species in these regions will not have areas available that present suitable climatic conditions. At the same time, a northward expansion of the distribution of *L. speciosa* may be expected, since it may be favored by the higher altitudes of the Sierra Madre Occidental mountains (>2800–3000). In these areas, mountains of similar elevations are relatively close to the current populations, and this proximity could favor colonization by the species.

The prediction of an upward shift in the distribution of *L. speciosa* of around 300–400 m, can be related to the increase in temperature in the lowest parts of the mountainous areas where it is currently distributed. At higher altitudes, lower temperatures and higher humidity may be climatically favorable for *L. speciosa*, which depends on rainfall and moisture. The shift of the species towards higher altitudes would also serve to avoid strong water stress, since these species are very sensitive to prolonged periods of drought^42,85,86^.

Although the models predicted the colonization of *L. speciosa* towards higher altitudes, this result must be considered with caution due to the possible effects of climate change on other interactions not considered in our study, such as pollination. Only two pollinator species are known for this species: *Bombus pennsylvanicus sonorus* Say and *Bombus ephippiatus* Say^87^ and, while *L. speciosa* might be able to disperse quickly enough to track adequate climatic conditions, a climate change-driven spatial or temporal imbalance could develop between the species and its pollinators^23^.

Other important factors affecting the distribution of this species that were not considered in this study are its capacities for dispersion and adaptation. Dispersal limitation may prevent the species from migrating at the same pace as climate changes, while the species could also adapt to the new climate, which would hamper our projections. However, even if such adaptation occurs for this species, it may not happen for the other species with which it interacts (e.g. host, pollinators, and mycorrhizae). These factors, along with the strict dependence of *L. speciosa* on *Q. deserticola* that indicates an incapability of adaptation to new host species, may potentially act to prevent the colonization of new areas^83,88^. In this context, we consider that, for species that are highly dependent on specialized interactions, exclusion of distribution information pertaining to the interacting species could lead to underestimation of the extinction risks faced by the species^88,89^. Despite the importance of our results, they must also be interpreted with caution because our approach is static and reflect only a snapshot of what occurs with the species in a specific moment in time^11,76,90,91^. Moreover, due to the complexity of biotic interactions and the limited nature of the pertinent knowledge, incorporation of this information into niche models continues to be a challenge^92^. Advancement in this field would allow the integration of solid bases for the incorporation of biotic predictors into the ENMs. This would result in models that reflect the dynamics of the species and produce more accurate estimates.

### Conservation status

*L. speciosa* is considered one of the most wild-harvested orchids in Mexico^39,93^, due to its widespread use for ornamental and cultural purposes^39,42^. Currently, this species is included in the Mexican Red List under the category “*Special protection*”^40^ and it is not listed in the IUCN. However, our results indicate that mainly because the elevated extraction of individuals of and the loss of its habitat, the species should be moved to “Threatened” and “*Vulnerable*” species in the Mexican and IUCN red list, respectively^43^.

Our results also could help to identify regions where the species may require the implementation of conservation programs. Habitat loss and intensive extraction of *L. speciosa* as a non-timber forest resource have led the species to a critical conservation status^41,94^. This is the case in the states of central Mexico, where thousands of individuals are wild-harvested and oak forests are being transformed into agricultural land, such as avocado plantations, especially in Michoacan state^39,95^.

The high exploitation of the oak forests^96,97^ has important implications in some populations of *L. speciosa*, causing the fragmentation and loss of populations. It is thus important to protect the habitat of this species, since small changes in the habitat can have profound consequences for its conservation^98–100^. Moreover, less than 0.5% of the distribution of *L. speciosa* falls within protected areas, in the biosphere reserve “*Barranca de Metztitlán*” in Central Mexico. It is therefore urgently required to detect important areas for conservation of the species, and this study provides a novel approach to better project its current and future distribution.

## Supplementary information

Supplementary Information.

## Data Availability

Environmental layers used as predictor layers in this study are publicly available and sourced in the main text of this paper. All occurrence records used to generate the ENMs in this study (orchid and host tree) are publicly available in herbarium collections or publications. The authors can provide vector resources of ENMs of the species models generated in this study if requested.
