# Supplementary material for: Distribution and conservation of species is misestimated if biotic interactions are ignored: the case of the orchid Laelia speciosa
Source: Sci Rep. 2020 Jun 12;10:9542. doi: 10.1038/s41598-020-63638-9 (PMC7293343; doi:10.1038/s41598-020-63638-9)
Supplement: Supplementary file 1 — Supplementary Information. [file 41598_2020_63638_MOESM1_ESM.docx]

**Distribution and conservation of species is misestimated if biotic interactions are ignored: the case of the orchid *Laelia speciosa***

Mayra Flores-Tolentino^1,3^, Raúl García-Valdés^2^, Cuauhtémoc Saénz-Romero^1^, Irene Ávila-Díaz^3^, Horacio Paz-Hernández^4^ and Leonel Lopez-Toledo^1^

Corresponding author: Leonel Lopez-Toledo, Instituto de Investigaciones sobre los Recursos Naturales, Universidad Michoacana de San Nicolás de Hidalgo, Av. San Juanito Itzícuaro s/n, Col. Nueva Esperanza, Morelia, Michoacán, CP 58330, Mexico. Email: [llopezt@umich.mx](mailto:llopezt@umich.mx), [leonellopeztoledo@gmail.com](mailto:leonellopeztoledo@gmail.com)

^1^Instituto de Investigaciones sobre los Recursos Naturales, Universidad Michoacana de San Nicolás de Hidalgo, Av. San Juanito Itzícuaro s/n, Col. Nueva Esperanza, Morelia, Michoacán, CP 58330, Mexico.

^2^CEFE UMR 5175, CNRS – Université de Montpellier – Université Paul-Valéry Montpellier – EPHE, F-34293, Montpellier cedex 5, Montpellier, France.

^3^Facultad de Biología, Universidad Michoacana de San Nicolás de Hidalgo, 48020 Morelia, Michoacán, Mexico.

^4^Instituto de Investigaciones en Ecosistemas y Sustentabilidad, Universidad Nacional Autónoma de México Unidad Morelia, Antigua Carretera a Pátzcuaro 8701 58190, Morelia, Michoacán, Mexico.

**Methods**

***Spatial autocorrelation for records***

The spatial autocorrelation of the presence data of the two species modeled in this study (*Quercus deserticola* and *Laelia speciosa*) was eliminated through a pattern analysis. This method first proves randomness of records for each species^1^. If the records pass the randomness test, 75% of the records are used for training the model and 25% for model validation. Second, if the records are not distributed randomly, a pattern analysis was applied to the records by estimating the distance at which it is possible to find a single species record with a maximum probability. The pattern analysis was performed by using the public domain ILWIS 3.7 (<http://52north.org/ilwis>). This analysis is similar to estimating the distance (or the range value of a variogram) for which the records do not show spatial autocorrelation^2^. Third, the distance obtained in the previous step is used to generate a grid cell that divides the study area. The estimated distance value expressed in degrees. Such a grid cells system is obtained by using the public Quantum GIS 3.4.0 software (http://qgis.osgeo.org). Finally, after the species records are randomly selected, a single record per grid cell is used to train the model.

***Ecological niche modeling of the host tree***

The modeling process for the host tree (*Quercus deserticola*) was followed the same procedure as the climate model of *Laelia speciosa*. In a general way it consisted of:

1.- Eliminate the autocorrelation of climatic variables with the Pearson test.

2.- The collinearity between the points of presence was eliminated with the pattern analysis.

3. The model was run in Maxent with the following configuration: 75% of the uncorrelated records were used to run the model and 25% for validation, with. the following parameters: maximum interactions (500), a convergence threshold (0.00001) and the maximum number of background points was 10,000. In order to avoid overfitting of the test data, we set the regularization multiplier value as 1.

***Multivariate environmental similarity surface (MESS) analysis***

The MESS analysis gives us the chance of mapping in the geographic space, the results of the comparison of climatic spaces^3^. We used the MESS analysis, integrated in Maxent^4^, which represents how similar a point is to a reference set of points, with respect to a set of predictor variables. The negative values show sites where at least one variable has a value that is outside the range of environments over the reference set, so these are novel environments. The values in the MESS are influenced by the full distribution of the reference points, so that sites within the environmental range of the reference points but in relatively unusual environments will have a smaller value than those in very common environments. Maxent provide two maps as a result of the MESS; in the first map, shown areas in red present one or more variables outside the range present in the training data (current range). The second map shows the most dissimilar variable (MoD), that is, the one that is furthest outside its training range^3^.


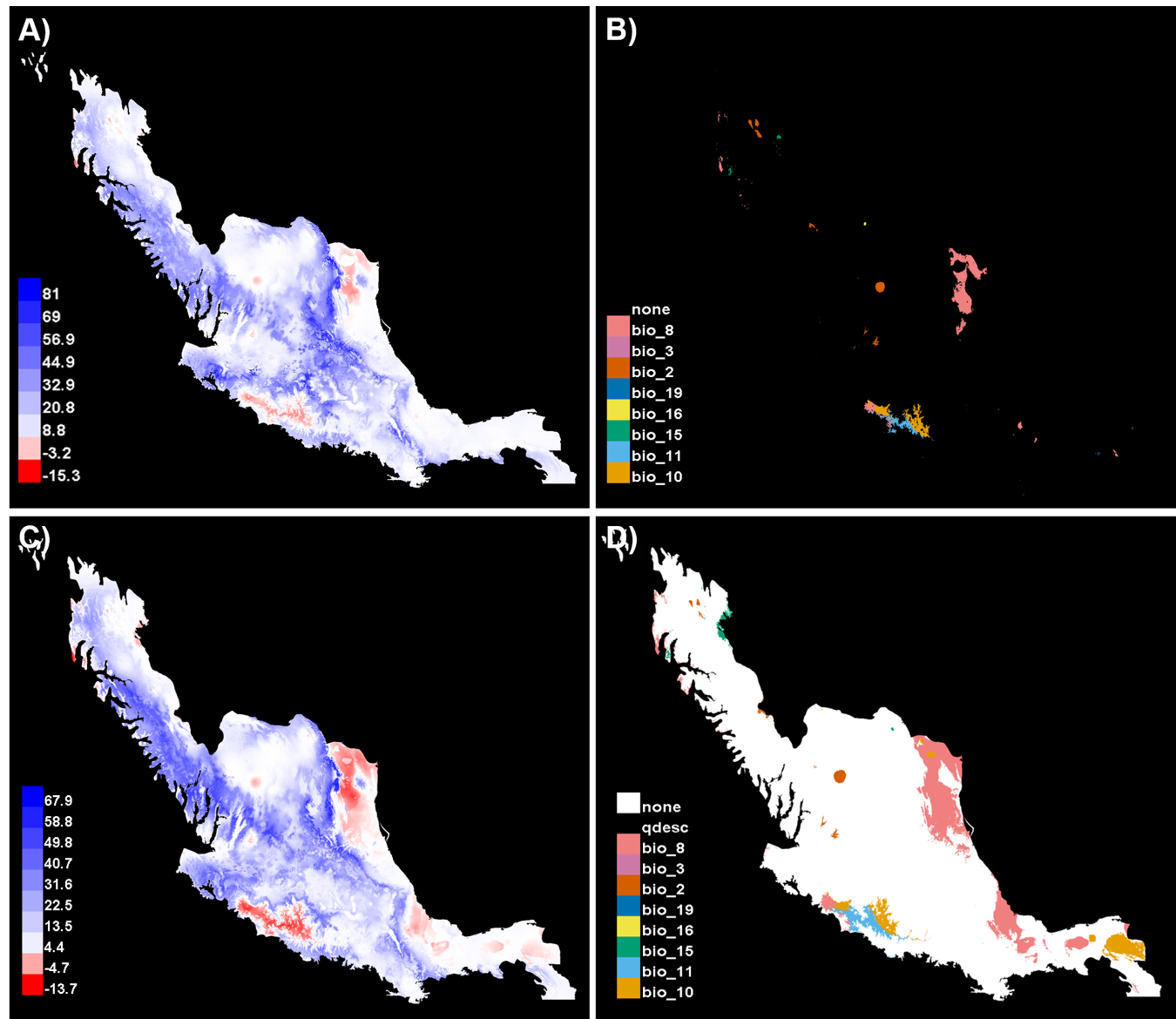


Figure SI1. Multivariate environmental similarity surface (MESS) analysis for Maxent climate models (A, B) and biotic models (C, D). Areas in the invasive range with climatic conditions similar to the native niche (positive values) are represented in blue. Increasingly novel (dissimilar) climatic conditions relative to the native niche (negative values) are indicated by the red gradient (A, C). MoD shows the climatic variables which present the higher differences between the native and invaded ranges. In our case, the main climatic difference between the native and invaded ranges locates in the northeast area of the study area, and it is given by Bio_08 (mean temperature of wettest quarter) shown in pink (B, D).

Table SI1. Climatic variables obtained from Worldclim (www.worldclim.org) and used in the modeling of *Laelia speciosa* and *Quercus deserticola.*

| **Variables** | ***Laelia speciosa*** | ***Quercus deserticola*** |
| --- | --- | --- |
| BIO1 = Annual Mean Temperature |  |  |
| BIO2 = Mean Diurnal Range (Mean of monthly (max temp - min temp)) |  |  |
| BIO3 = Isothermality (BIO2/BIO7) (* 100) |  |  |
| BIO4 = Temperature Seasonality (standard deviation *100) |  |  |
| BIO5 = Max Temperature of Warmest Month |  |  |
| BIO6 = Min Temperature of Coldest Month |  |  |
| BIO7 = Temperature Annual Range (BIO5-BIO6) |  |  |
| BIO8 = Mean Temperature of Wettest Quarter |  |  |
| BIO9 = Mean Temperature of Driest Quarter |  |  |
| BIO10 = Mean Temperature of Warmest Quarter |  |  |
| BIO11 = Mean Temperature of Coldest Quarter |  |  |
| BIO12 = Annual Precipitation |  |  |
| BIO13 = Precipitation of Wettest Month |  |  |
| BIO14 = Precipitation of Driest Month |  |  |
| BIO15 = Precipitation Seasonality (Coefficient of Variation) |  |  |
| BIO16 = Precipitation of Wettest Quarter |  |  |
| BIO17 = Precipitation of Driest Quarter |  |  |
| BIO18 = Precipitation of Warmest Quarter |  |  |
| BIO19 = Precipitation of Coldest Quarter |  |  |


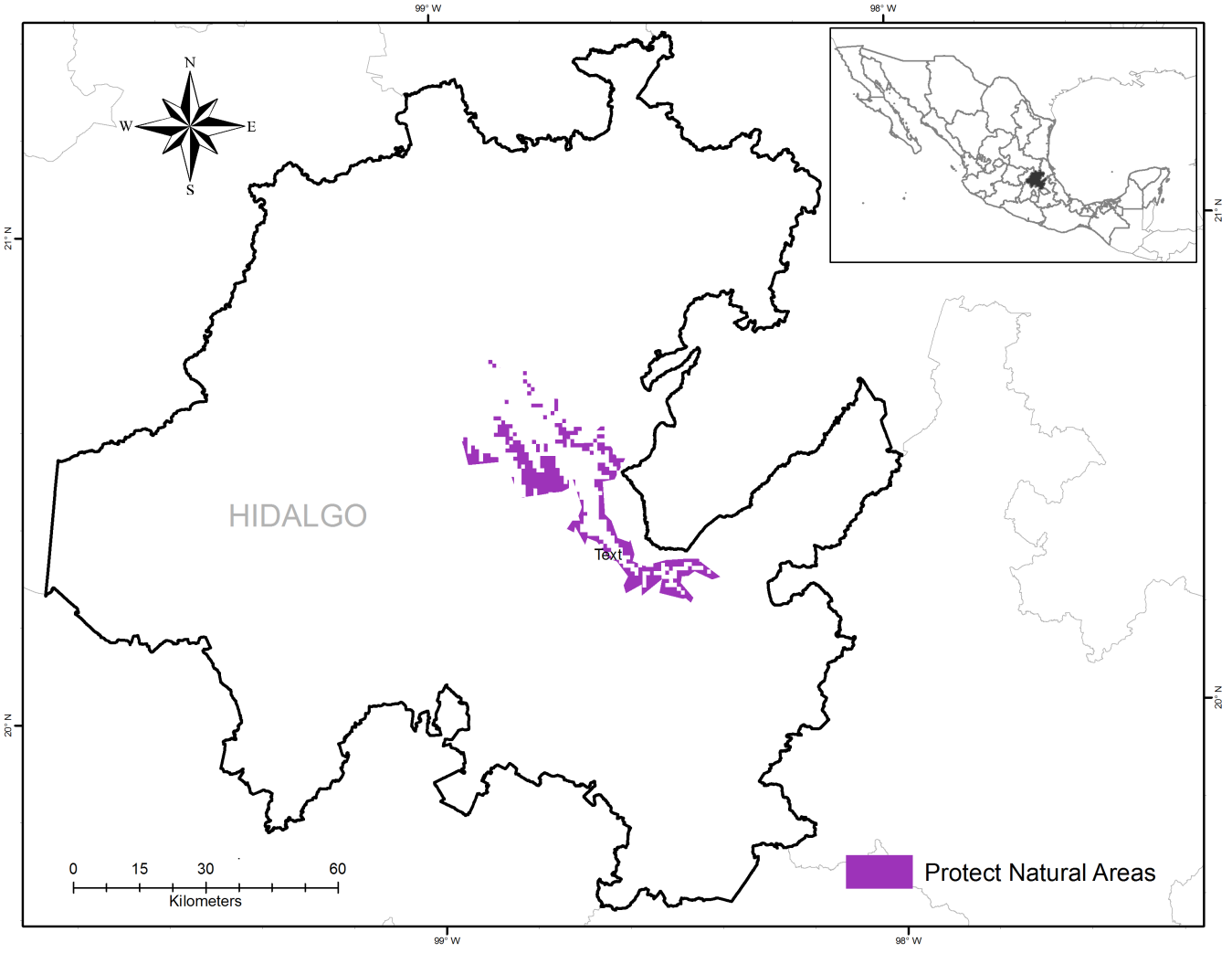


Figure SI2. Maps illustrate the Protected Natural Areas where *Laelia speciosa* has been registered in Mexico.

**References**

1. Bivand, R. S., Pebesma, E. J. & Gómez-Rubio, V. Applied spatial data analysis with R. Spring, New York (2008).
2. Hengl, T. A practical guide to geostatistical mapping of environmental variables. European Commission, Joint Research Centre, Institute for Environment and Sustainability, Italy (2007).
3. Elith, J., Kearney, M. & Phillips, S. The art of modelling range‐shifting species. *Methods* *Ecol Evol*, 1, 330–342. https://doi.org/10.1111/j.2041-210X.2010.00036.x (2010).
4. Phillips, S. J., Dudík, M. & Schapire, R. E. Maxent software for modeling species niches and distributions (2017).
